# Associations of Apoptotic and Anti-Apoptotic Factors with Beef Quality, Histochemical Characteristics, and Palatability of Hanwoo *Longissimus thoracis* Muscle

**DOI:** 10.3390/ani12040467

**Published:** 2022-02-14

**Authors:** Boin Lee, Jae-Yeong Kim, Young-Min Choi

**Affiliations:** Department of Animal Sciences and Biotechnology, Kyungpook National University, Sangju-si 37224, Korea; ananassab@knu.ac.kr (B.L.); rlarbgml0603@naver.com (J.-Y.K.)

**Keywords:** tenderness, apoptosis, caspases, cytochrome c, small heat shock proteins, Hanwoo

## Abstract

**Simple Summary:**

After the onset of muscle fiber apoptosis, various apoptotic and anti-apoptotic factors are selectively increased. These factors are directly and/or indirectly associated with tenderness variation. Given the complex relationship between the apoptosis-related molecules, more data are needed on how the expression patterns of these factors during the post-mortem period affects the palatability of beef. In this study, the combination of expression levels of apoptotic (cytochrome c and caspases) and anti-apoptotic (small heat shock proteins) factors at 45 min post-mortem tended to be associated with Warner-Bratzler shear force and tenderness attributes in Hanwoo steers with a lower degree of marbling. Meanwhile, levels of these molecules at 24 h had a somewhat limited effect on the beef tenderness. Clearly, these results demonstrate that expression levels of apoptotic and anti-apoptotic molecules at the early post-mortem period may be a relevant indicator to explain the tenderness variation in Hanwoo.

**Abstract:**

This study compared the meat quality, histochemical traits, palatability, and expression levels of apoptotic (cytochrome c and caspases) and anti-apoptotic (small heat shock proteins) factors at 45 min and 24 h post-mortem of Hanwoo *Longissimus thoracis* muscles in groups categorized by Warner-Bratzler shear force (WBS) values to investigate the association between beef tenderness variation and apoptosis-related molecules. There were no differences in marbling scores, meat quality traits, or histochemical characteristics among the WBS groups (*p* > 0.05) indicating no significant effect on the tenderness variation in the current study. On the other hand, the low group exhibited higher levels of apoptotic and anti-apoptotic factors (except for αβ-crystallin) at 45 min post-mortem compared to the high WBS group, resulting in higher scores of tenderness attributes (*p* < 0.05). However, the level of αβ-crystallin at 45 min post-mortem was lower in the low and medium WBS groups compared to the high WBS group (*p* < 0.0106). At 24 h post-mortem, no significant differences were observed in the expression levels of apoptosis-related factors among the WBS groups (*p* > 0.05) except for heat shock protein 27 (*p* < 0.05).

## 1. Introduction

Among the various eating-quality characteristics of cooked beef, tenderness is one of the primary attributes that consumers most value, as it plays an important role in determining the overall acceptance of the meat [1]. Thus, an ongoing aim of the beef industry is to ensure that the tenderness of beef, as experienced by consumers, is consistent and/or improving [2,3]. To improve beef tenderness, it is necessary to understand the biochemical and physical changes of skeletal muscle during the post-mortem period [4]. After exsanguination, muscle fiber apoptosis (a highly organized cell death program) is initiated, and the expression of various apoptotic and anti-apoptotic molecules, including calpains, caspases, and heat shock proteins (HSPs), is intrinsically and selectively increased [5]. Expression levels of these proteins are directly and/or indirectly associated with the degradation of myofibrillar proteins and responsible for variation in beef tenderness [1]. Among these proteins, calpain, a well-known Ca^2+^-dependent cysteine protease, has been most thoroughly investigated in terms of its relationship with tenderness [6,7]. However, the calpain system cannot fully explain all the variations in beef tenderness [8]; thus, additional research on the other proteins associated with cell apoptosis during the post-mortem period is needed.

Caspases, the family of cysteine aspartate-specific proteases, are responsible for proteolytic cleavage leading to apoptotic cell death in various tissues [6,7,8]. Fourteen caspases have been identified, and these proteases can be divided into two subgroups—initiator and executioner caspases—according to their signaling pathways [9]. Caspase-mediated apoptosis initially occurs via the release of cytochrome c, which acts as a trigger from mitochondria to the cytoplasm [10]. In the cytoplasm, elevated cytochrome c levels can activate the initiator caspases, especially caspase 9, and activated initiators lead to increased expression levels of effector caspases, including caspases 3 and 7 [9]. Meanwhile, HSPs are also synthesized and expressed, and can play a variety of roles during apoptosis as molecular chaperones [11]. In particular, small HSPs, including αβ-crystallin, HSP20, and HSP27, can interfere with the recruitment of procaspases and proteolytic activation of caspases, consequently delaying the onset of apoptosis [11,12]. In contrast, these HSPs can facilitate proteolysis via endogenous proteases by stabilizing unstable proteins and inhibiting the formation of aggregated proteins [13].

Considering the complex relationship between the apoptotic and anti-apoptotic proteins, more data on the combination of their protein expression levels and on individual expression levels during the post-mortem period is required; furthermore, how these expression levels affect the palatability, especially tenderness, of beef should be investigated. Therefore, the objective of the present study was to determine the effects of cytochrome c, caspases (caspases 3, 7, and 9), and small HSPs (αβ-crystallin, HSP20, and HSP27) expression levels during the post-mortem period on the meat quality, histochemical, and eating quality characteristics of Hanwoo *Longissimus thoracis* (LT) muscle groups according to Warner-Bratzler shear force (WBS) values. Overall, the aim was to understand the association between variation in beef tenderness and apoptosis-related proteins.

## 2. Materials and Methods

### 2.1. Animals and Muscle Samples

The Hanwoo steers were obtained at the local slaughterhouse (Hoengseong, South Korea) in eight batches (five to six steers per batch with 41 steers in total). Following the standard slaughter procedures of the Korea Institute for Animal Products Quality Evaluation (KAPE) [14], 41 LT muscles (approximately 800 to 1000 g per animal) were used in the present study. At 45 min post-mortem, the pH value of each LT muscle was measured at the same location, i.e., at 13th thoracic vertebra (the standard location for measurement of beef quality grades). Additionally, muscle samples (about 40 g per sample) were collected from the midsection of the LT muscle at the 13th thoracic vertebra, frozen by liquid nitrogen, and then stored at −80 °C for histochemical analysis, quantitative real-time polymerase chain reaction (RT-PCR), and Western blot analysis. At 24 h post-mortem, a quality grading evaluation, including marbling and texture, was performed according to the carcass grading standard of the KAPE [14]. Marbling was graded on a 9-point scale (devoid to very abundant) by trained evaluators of the KAPE [14], where the loin samples used in this study had beef marbling standard scores of 1–5. After grading of the carcass quality, samples were obtained between the 9th to 13th thoracic vertebrae of the LT muscles to be used for measurements of meat quality, including WBS, and eating quality characteristics. Meat quality characteristics, including WBS, muscle pH, meat color, and water-holding capacity, were immediately assessed in cold room (4 °C). The samples were then removed into steak-size cuts (1.5 cm thick; about 100–120 g per sample) and were stored at –20 °C until sensory quality analysis. The intramuscular fat (IMF) content was determined using the Soxhlet method with a solvent extraction system [15].

### 2.2. Meat Quality Characteristics

At 24 h post-mortem, meat quality traits were measured. WBS analysis was conducted according to a previously published method [16]. Each beef sample (approximately 80 g) at 24 h post-mortem was put into a polyethylene bag, and then heated in a temperature-controlled water bath (80 °C) until the core temperature reached 71 °C according to a spear-type thermometer (Testo 108, Testo Inc., Lenzkirch, Germany) measurement. Cooked samples were cooled in an ice-slurry until equilibration, after which at least 10 core samples (1.27 cm diameter) were produced by cutting parallel to the muscle fiber orientation. Core samples were analyzed using an Instron Universal Testing machine (Model 1011, Instron Corp., Canton, MA, USA) arraying a Warner-Bratzler blade operating at a crosshead speed of 200 mm/min. 

The pH_24 h_ of each LT muscle was measured using a portable pH- and temperature-measuring instrument with a penetration probe (Testo 206-pH2, Testo AG, Lenzkirch, Germany). After 30 min of blooming time at 4 °C, meat surface color was assessed using a Minolta chromometer (diffuse D65; illumination C; viewing angle, 0°; port/viewing area, 8 mm; CR-400, Minolta Camera Co., Osaka, Japan). Color values, including lightness (L*), redness (a*), and yellowness (b*), were measured according to the recommendations of the Commission Internationale de l’Eclairage [17]. To measure drip loss [16], muscle samples (initial weight of about 80 g) were placed inside plastic bag in such a way as to ensure that they were not attached to the plastic bag, after which they were hung on nets in a cold room (4 °C) for 48 h. Drip loss percentage was calculated as the difference in sample weight before and after 48 h. Filter-paper fluid uptake (FFU) was measured by pre-weighing filter paper, placing it onto the meat surface for less than 2 s to absorb fluids, and then weighing it again [18]. The preparation of cooked meat samples for cooking loss was identical to that used for WBS analysis, and cooking loss was calculated by weighing the samples before and after cooking [16].

### 2.3. Histochemical Analysis

Serial muscle cross-sections were obtained using a cryostat (CM1860, Leica Biosystems, Wetzlar, Germany) set at 10 μm thickness and –25 °C. To measure fiber and bundle characteristics, muscle sections were stained using the myosin ATPase staining method with acid-preincubation (pH 4.3). Muscle fiber types, including I, IIA, and IIX, were classified according to their contractile and metabolism characteristics [19]. Stained samples were photographed through a microscope (DM500, Leica Microsystems, Wetzlar, Germany) equipped with a high-definition digital camera (ICC50, Leica Microsystems, Milton Keynes, England). Using Image-Pro Plus software (Media Cybernetics, Silver Spring, MD, USA), the histochemical images were analyzed, and more than 600 fibers and 30 bundles were used for statistical analysis of histochemical traits. To assess fiber characteristics, mean area, total fiber number, and fiber area percentage were measured at 100× magnification. The mean fiber area was determined as the total area divided by the total number of fibers counted. Fiber density was calculated from the average fiber number per mm^2^ (data not shown). The total fiber number was determined by multiplying muscle fiber density by the loin-eye area. Area percentage of each fiber type was calculated as the ratio of the total area of each fiber type to the total area measured. For bundle characteristics, bundle area and fiber number per bundle were measured at 40× magnification.

### 2.4. Sensory Evaluation

A total of 41 LT samples at 24 h post-mortem were randomly selected by coding with a 3-digit number and evaluated twice during 16 sessions (5–6 samples per session). The panelists (six women and five men aged 23–48 years) were trained for at least 6 months at the Muscle Biology Laboratory of Kyungpook National University (KNU 2019–0027) according to the guidelines of the American Meat Science Association [20]. Frozen LT samples were thawed at 4 °C for 18 h, after which they were cooked by pan-frying using an induction electric range until their core temperature reached 71 °C (CIR-IH300RGL, Cuchen, Cheonan, Korea). Trained panelists were provided with cooked samples (1.3 cm^3^ cubes). During the sensory evaluation, panelists were served water and non-salt crackers to refresh their mouths before and after the evaluation of each sample. The following sensory quality traits were assessed on 9-point scale (1 to 9; low to high): softness (very hard to very soft), initial tenderness (very tough to very tender), chewiness (very chewy to very tender), rate of breakdown (very slow to very fast), amount of perceptible residue (highly abundant to none), overall tenderness (dislike extremely to like extremely), juiciness (not juicy to extremely juicy), flavor intensity (very weak to very strong), off-flavor intensity (very strong to very weak), and overall acceptability (dislike extremely to like extremely).

### 2.5. Quantitative RT-PCR

Total RNA was isolated according to the manufacturer’s instructions and then RNA quantity was measured using a real-time PCR instrument (ABI 7300, Applied Biosystems, CA, USA). The quality of RNA was evaluated by gel electrophoresis and normalized accordingly. One nanogram of total RNA was used to synthesize complementary DNA. Quantitative RT-PCR was used to determine the relative mRNA expression levels of *CYCS* (cytochrome c), *CASP 9* (caspase 9), *CASP 3* (caspase 3), *CASP 7* (caspase 7), and *GAPDH* (glyceraldehyde-3-phosphate dehydrogenase) in LT muscle. The sequences of *CYCS*, *CASP 9*, *CASP 3*, *CASP 7*, and *GAPDH* were as follows: 5′-GAA TGG GTG TCC GCA ACG-3′, 5′-TTG GCA CAA GAG CAG TCG TT-3′; 5′-TTG ACC CAT CAA AGC CGA GC-3′, 5′-ACC TCT GGT CTG AGA ACC TCA-3′; 5′-GAA CTT CCA CGA AAA TAC TGG CA-3′, 5′-TCC TGA CTT CGT ATT TCA AGT TCA-3′; 5′-GAA TGG GTG TCC GCA ACG-3′, 5′-TTG GCA CAA GAG CAG TCG TT-3′; 5′-CCT GCC CGT TCG ACA GAT AG-3′, and 5′-AGT GAA GAC CCC AGT GGA CT-3′, respectively. Quantitative RT-PCR was performed using SYBR green dye (A25741, Applied Biosystems) and a real-time PCR instrument (ABI 7300, Applied Biosystems). Relative gene expression was calculated using the comparative 2^−ΔΔCt^ method for relative quantification.

### 2.6. Western Blot Analysis

Muscle proteins were extracted from homogenizing LT muscles (about 0.12 g) in radio immunoprecipitation assay buffer (1.2 mL) at 45 min and 24 h post-mortem. Proteins were quantified and normalized using Coomassie staining. Muscle proteins (5 μL) were separated through 12% SDS-PAGE gel using a Mini-PROTEAN system (Bio-Rad Laboratories Inc., Richmond, CA, USA) and then transferred to polyvinylidene difluoride membranes (GE Healthcare Ltd., Freiburg, Germany). Transferred membranes were blocked with 5% non-fat dry milk powder in Tris-buffered saline/Tween. For Western blot analysis, the following primary antibodies were used: αβ-crystallin (1:10,000 dilution; ab13496, Abcam Ltd., Cambridge, UK), HSP20 (1:1000 dilution; ab13491, Abcam Ltd.), and HSP27 (1:3000 dilution; sc-13132, Santa Cruz Biotechnology Inc., Santa Cruz, CA, USA). HSP27 antibody detects 27 kDa (intact form) and 22 kDa (degraded form) proteins. The secondary antibody used was anti-mouse immunoglobulin G (IgG) horseradish-peroxidase-linked antibody (1:3000 dilution; Cell Signaling Technology Inc., Danvers, MA, USA). A WesternBright ECL Kit (Advansta Inc., Menlo Park, CA, USA) was used to detect proteins and images were taken using the ImageQuant LAS 500 (GE Healthcare Ltd., Freiburg, Germany). Each protein band was analyzed using one-dimensional image analysis software (Eastman Kodak Co., Rochester, NY, USA).

### 2.7. Statistical Analysis

The FASTCLUS procedure (SAS Institute, NC, USA) was performed to generate three clusters based on WBS values (low, n = 14; medium, n = 14; high, n = 13). Regarding WBS values, meat quality traits, histochemical traits, and the expression levels of cytochrome c, caspases, and small HSPs, a general linear mixed model (GLM) procedure was conducted to elucidate any associations. Regarding the sensory quality characteristics, a GLM was produced by analyzing the WBS value as a fixed effect and the trained panelists as a random effect. Significant differences in the least-square means (LSMs) for investigated parameters between the groups were compared through the probability difference option at *p* < 0.05. All data are presented as LSMs with standard errors.

## 3. Results

### 3.1. Comparison of Meat Quality and Histochemical Characteristics 

As expected, a marked difference was detected in the WBS values of the LT muscle among the groups (Table 1); the WBS values were appropriately higher in the order of the high, medium, and low groups (67.4 vs. 58.9 vs. 50.9 N, *p* < 0.0001). No differences were observed in the marbling score and IMF content among these groups (*p* > 0.05), and similar texture and firmness scores, as measured by trained carcass evaluators, were observed among the groups (data not shown, *p* > 0.05). Regarding the meat quality traits, beef samples did not differ in terms of muscle pH at 45 min and 24 h post-mortem among the groups (*p* > 0.05). Measured lightness, redness, and yellowness values were similar among the groups (*p* > 0.05). Additionally, the WBS value groups exhibited no differences in water-holding capacity parameters, including drip loss, FFU, and cooking loss (*p* > 0.05). 

There were no significant differences in fiber area, total fiber number, or fiber composition among the groups (Table 2; *p* > 0.05). Moreover, the mean area and fiber number per bundle did not differ significantly among the groups (*p* > 0.05).

### 3.2. Comparison of Eating Quality Characteristics

All tenderness attributes differed significantly between the low and high groups (Table 3; *p* < 0.05). Higher scores for softness (6.02 vs. 5.20, *p* = 0.0012) and initial tenderness (5.92 vs. 5.00, *p* = 0.0016) were observed when comparing the low group to the medium group; however, there was no difference in these scores between the medium and high groups (*p* > 0.05). The medium group also had similar scores for chewiness, rate of breakdown, and amount of perceptible residue relative to the scores for the other groups (*p* > 0.05), whereas significantly lower scores for these traits were observed for the high group relative to the scores recorded for the low group (*p* < 0.05). Like softness, the low group had a higher acceptability of overall tenderness compared to the medium and high groups (5.78 vs. 4.94 and 4.47, *p* = 0.0037). No differences were observed in juiciness, flavor intensity, or off-flavor intensity among the groups (*p* > 0.05). LT steaks from the low group showed a higher overall acceptability compared to LT steaks from the medium and high groups (5.71 vs. 4.93 and 4.49, *p* = 0.0065).

### 3.3. Comparison of Apoptotic and Anti-Apoptotic Factors 

**Figure 1:**
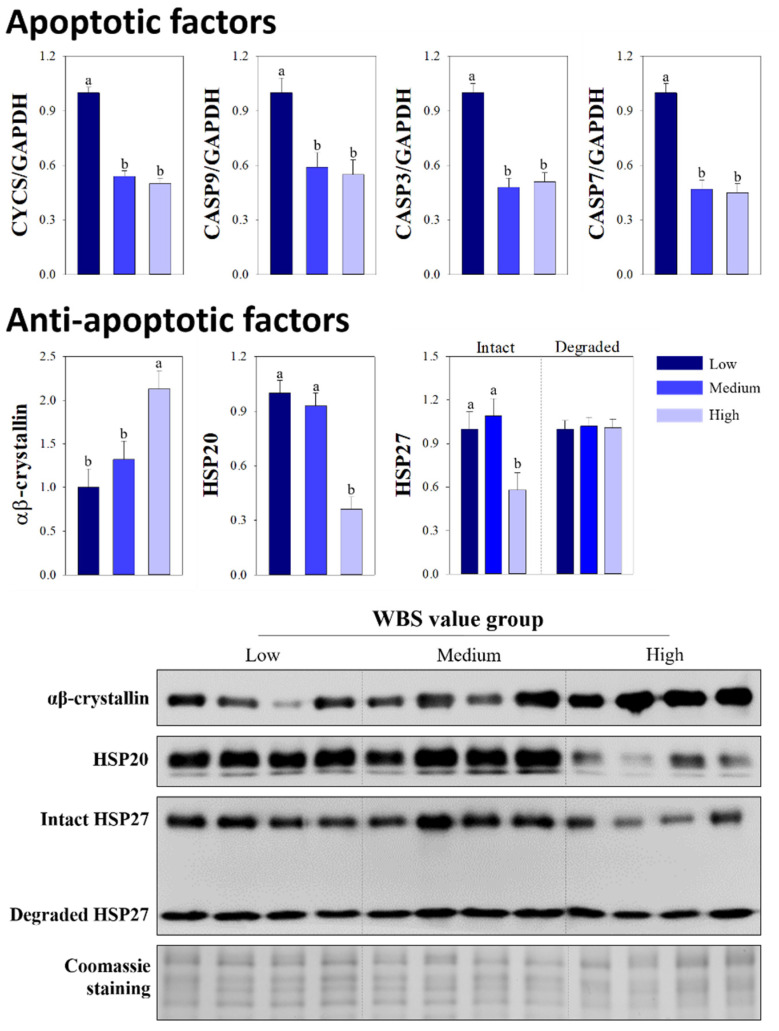
Quantitative RT-PCR for expression of cytochrome c (*CYCS*), caspase 9 (*CASP 9*), caspase 3 (*CASP 3*), and caspase 7 (*CASP 7*), and the relative intensities and Western blot images of the indicated heat shock proteins (HSPs) in the Hanwoo *Longissimus thoracis* muscle at 45 min post-mortem for groups categorized by Warner-Bratzler shear force (WBS) values. Gels stained by Coomassie blue were used as protein loading controls. Bars indicate the standard error of the mean. ^a,b^ Different letters denote significant differences (*p* < 0.05).

At 45 min post-mortem, higher levels of the apoptotic factors, including cytochrome c and caspases 9, 3, and 7, were observed in the low group compared to the medium and high groups (Figure 1; *p* < 0.05), whereas such differences were not detected between the medium and high groups (*p* > 0.05). The high group showed a higher level of αβ-crystallin compared with the levels detected in the low and medium groups (2.13 vs. 1.00 and 1.32, *p* < 0.0106); whereas, the high group had a lower level of HSP20 compared to the other groups (*p* < 0.0003). Higher expression levels of intact HSP27 were detected in the low group compared with levels in the high group (1.00 vs. 0.58, *p* < 0.0286), however, the levels of degraded HSP27 were similar among the WBS value groups (*p* > 0.9803). 

**Figure 2:**
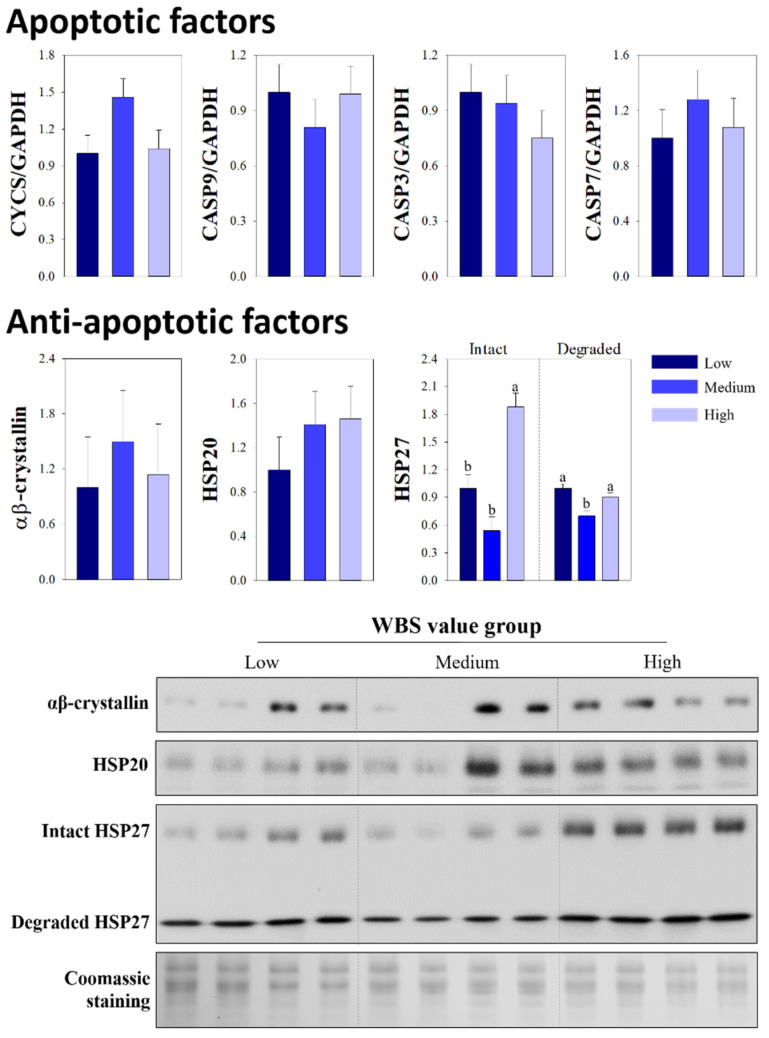
Quantitative RT-PCR for expression of cytochrome c (*CYCS*), caspase 9 (*CASP 9*), caspase 3 (*CASP 3*), and caspase 7 (*CASP 7*), and the relative intensities and Western blot images of the indicated heat shock proteins (HSPs) in the Hanwoo *Longissimus thoracis* muscle at 24 h post-mortem for groups categorized by Warner-Bratzler shear force (WBS) values. Gels stained by Coomassie blue were used as protein loading controls. Bars indicate the standard error of the mean. ^a,b^ Different letters denote significant differences (*p* < 0.05).

At 24 h post-mortem, the levels of all apoptotic factors were similar among the groups (*p* > 0.05; Figure 2). Unlike at 45 min post-mortem, similar levels of αβ-crystallin and HSP20 were detected among the WBS value groups (*p* > 0.05). However, the low group exhibited a lower level of intact HSP27 compared with that in the high group (*p* < 0.0122), although similar levels of degraded HSP27 were observed in the low and high groups (*p* > 0.05).

## 4. Discussion

The tenderness of beef steers is substantially important to consumer acceptance and satisfaction [21]. Tenderness is known to vary due to several intrinsic and extrinsic factors [22]; it is commonly related to the characteristics of the major components of skeletal muscle including the connective tissue, IMF, and histochemical characteristics [23]. Numerous studies have reported negative correlations between WBS values and the degree of marbling, which is an integral part of beef grade assessment, since the IMF content can be associated with the textural properties (especially hardness) and water retention of cooked beef [24,25]. Previously, Choi et al. [26] confirmed that muscle fiber bundle traits can affect the texture and firmness of muscle surface. Moreover, the WBS values are positively related to the bundle size or fiber number per bundle in Hanwoo LT muscle [23,27], and showed a strong negative correlation with tenderness attributes performed by trained panelists [26]. In the present study, the WBS values of all groups tended to be higher as the degree of marbling was lower than that of highly marbled beef in the previous study [23]. The WBS groups classified by cluster analysis did not differ in terms of marbling and texture scores; these groups were also similar in their meat quality and histochemical characteristics, including bundle traits. In contrast, significant differences were observed in the tenderness attributes, as assessed by trained panelists, of the low and high WBS groups, although the panelists did not recognize any differences in juiciness or flavor intensity between the two groups. These results suggest that the WBS value and tenderness variation in LT muscle samples at 24 h post-mortem were not caused by the degree of marbling or histochemical traits; indeed, this variation seems to be more related to other biochemical factors that affect tenderness in this study.

During the slaughtering and exsanguination processes, the skeletal muscle of livestock is deprived of oxygen and the muscle cells become ischemic [28]. Various biochemical and structural changes occur in the process of muscle conversion into meat including mitochondrial disruption, an end to ATP production, cytoplasmic acidification, and calcium deregulation in the cells [28]. These biochemical changes make cells prone to undergoing programmed cell death [29]. The activation of apoptosis proceeds according to an intrinsic or extrinsic pathway [30]; Ouali et al. [7] reported that apoptosis that manifests under ischemic conditions is activated via an intrinsic pathway. This cell apoptosis process is a mitochondrion-centered cell death that mediates outer membrane permeability and reduction in the inner transmembrane potential of mitochondria [9]. Due to these mitochondrial changes, the release of cytochrome c from the mitochondrial intermembrane space to the cytosol is increased, which in turn initiates the apoptotic cascade [30]. Elevated cytosolic cytochrome c can then bind apoptosis protease-activating factor 1 in the presence of dATP [30], and this binding form, known as the apoptosome, is essential for the activation of initiator and effector caspases [31]. In apoptotic muscle cells, released cytochrome c and activated caspases contribute to apoptosis-associated biophysicochemical changes [5]. In post-mortem cells, as the expression of apoptotic molecules such as cytochrome c and caspases increases, the extent of protein degradation and loss of cytoskeletal integrity also increases [5]. For these reasons, the positive effect of caspase proteolytic system on the tenderness of cooked pork, lamb, and beef has been reported in several studies, although the other endogenous proteolytic systems, such as calpain, are also attributed to tenderness variation after completion of rigor mortis [32,33,34,35]. Meanwhile, Underwood et al. [36] reported that caspase-3 activity did not differ between the beef steaks obtained from the low and high WBS groups. In this study, the caspase systems responsible for muscle fiber destruction during the apoptotic processes tended to be associated with the WBS variations after completion of rigor mortis. Beef steaks showing higher expression levels of apoptotic factors at the early post-mortem period exhibited significantly higher tenderness attribute values compared with those of steaks showing lower expression levels of apoptotic factors, although significant differences were not observed between the medium and high groups. Thus, expression levels of intrinsic apoptosis-related factors, including cytochrome c and caspases, at 45 min post-mortem can contribute directly and/or indirectly to the sensory tenderness attributes of Hanwoo cooked steaks. 

To maintain cell homeostasis, small HSPs protect the muscle fibers against the mitochondria-mediated apoptosis by blocking the release of cytochrome c, thereby reducing apoptosome formation and inhibiting caspase activities [6]. In particular, αβ-crystallin prevents irreversible damage to myofibrillar proteins by proteolysis [1,29]. In the present study, this anti-apoptotic function of αβ-crystallin may support the observed associations between HSPs and apoptotic factors at the early post-mortem period. Specifically, compared with muscle samples from the high WBS group, muscles from the low group exhibited lower expression levels of αβ-crystallin at 45 min post-mortem, which resulted in higher levels of apoptotic factors. Thus, overexpression of αβ-crystallin during the post-mortem period is associated with the toughness of cooked beef. Meanwhile, anti-apoptotic molecules are known to have diverse chaperoning functions during apoptotic processes [6,37]. Other small HSPs, including HSP20 and HSP27, can not only inhibit the activities of apoptotic factors, but also stabilize and prevent the aggregation of myofibrillar proteins which decreases their susceptibility to proteolytic degradation [38]. Thus, unlike αβ-crystallin, the increased expression levels of these proteins during apoptosis have been associated with the improved tenderness of cooked beef [29]. Here, in Hanwoo steers with a lower degree of marbling, similar result was observed; levels of HSP20 and intact HSP27 at 45 min post-mortem were significantly higher in the low and medium WBS groups than in the high WBS group. Unlike the intact form of HSP27, differences were not observed in levels of the degraded form between the WBS groups at 45 min post-mortem. Thus, the low group showing higher scores of tenderness attributes exhibited similar levels of all anti-apoptotic factors compared to the medium group, but the low group had higher levels of all apoptotic factors contributing to enzymatic protein cleavage than the medium group. These results indicated that the apoptotic potentials at 45 min post-mortem were different between the two groups. The difference in apoptotic potentials between the low and medium groups, which can be explained by the combination of different levels of apoptotic factors and similar levels of anti-apoptotic factors at the early post- mortem period, was the cause of tenderness variation.

Apoptosis-associated factors show significant changes in expression and activity levels during the post-mortem period with different temporal patterns [39,40]. For apoptotic molecules, the release of cytochrome c and caspases increases rapidly in the cytoplasm at the early post-mortem period [41], and small HSPs as anti-apoptotic factors are detected and show increased levels in myofibrils within 30 min after the onset of ischemic stress [29,38]. After the early post-mortem period, the expression levels of both apoptotic and anti-apoptotic factors gradually decrease, and the biological properties of these molecules are impaired during the post-mortem period [38,41]. For these reasons, the levels of apoptosis-related proteins at 24 h post-mortem had a limited effect on beef tenderness variations, unlike the effects of levels of these molecules at the early post-mortem period. Indeed, with the exception of HSP27 levels, differences were not observed among the groups in terms of the levels of either apoptotic or anti-apoptotic factors at 24 h post-mortem. However, observed differences in the levels of HSP27 among the groups at 24 h post-mortem seem not to directly affect variation in tenderness. Therefore, the apoptosis-mediated biochemical and physical changes that are associated with tenderness variation are mainly influenced by the expression levels of apoptotic and anti-apoptotic proteins at the early post-mortem period rather than during other periods.

## 5. Conclusions

Overall, the marked difference in the WBS values of cooked Hanwoo beef among the WBS groups was associated with expression levels of apoptotic and anti-apoptotic factors. Increased expression of apoptotic factors and decreased expression of anti-apoptotic factors at 45 min post-mortem was accompanied by a greater score of sensory tenderness in the low WBS group compared to the high WBS group. Moreover, the difference in the tenderness scores of cooked beef between the low and medium groups could be explained by the different apoptotic potentials at 45 min post-mortem. Thus, these molecules are particularly involved in the initial step of tenderization. Consequently, the levels of apoptosis-related molecules detected during the early post-mortem period could be considered relevant indicators that explain the tenderness variation in the Hanwoo LT muscles that have similar marbling scores and texture features.

## Figures and Tables

**Table 1:** Comparison of meat quality traits of the Hanwoo *Longissimus thoracis* muscle in groups categorized according to Warner-Bratzler shear force (WBS) values.

	WBS Value Group			*p*-Value
	Low (*n* = 14)	Medium (*n* = 14)	High (*n* = 13)	
WBS (N)	50.9 ^c^ (0.94) ^1^	58.9 ^b^ (0.94)	67.4 ^a^ (1.06)	<0.0001
Marbling score	3.93 (0.32)	3.79 (0.32)	3.67 (0.34)	0.9082
IMF content (%)	7.98 (1.63)	9.27 (1.63)	9.12 (1.63)	0.7642
Muscle pH_45 min_	6.14 (0.09)	6.22 (0.09)	6.12 (0.09)	0.7893
Muscle pH_24 h_	5.45 (0.04)	5.56 (0.04)	5.45 (0.04)	0.1467
Lightness (L*)	29.9 (1.07)	30.0 (1.07)	28.2 (1.16)	0.8546
Redness (a*)	16.1 (0.78)	16.3 (0.78)	9.06 (1.45)	0.9596
Yellowness (b*)	7.52 (1.34)	7.71 (1.34)	9.06 (1.45)	0.6560
Drip loss (%)	1.07 (0.16)	0.99 (0.16)	0.80 (0.17)	0.3913
FFU (mg)	3.12 (1.11)	3.11 (1.11)	5.57 (1.19)	0.9995
Cooking loss (%)	22.6 (2.42)	23.6 (2.42)	25.1 (2.61)	0.4044

^a–c^ Different superscript letters in the same row represent significant differences (*p* < 0.05). ^1^ Standard error of the mean. Abbreviations: IMF, intramuscular fat; FFU, filter-paper fluid uptake.

**Table 2:** Comparison of muscle fiber and bundle characteristics of the Hanwoo *Longissimus thoracis* muscle in groups categorized according to Warner-Bratzler shear force (WBS) values.

	WBS Value Group			*p*-Value
	Low	Medium	High	
Muscle fiber area (μm^2^)	4030 (236) ^1^	3995 (236)	4190 (226)	0.8304
Total fiber number (×1000)	2444 (185)	2335 (185)	2363 (151)	0.8896
Fiber area percentage (%)				
Type I	24.8 (1.65)	19.4 (1.65)	22.4 (1.34)	0.0610
Type IIA	23.7 (2.42)	25.2 (2.42)	25.7 (1.98)	0.7780
Type IIX	51.4 (2.60)	55.3 (2.60)	51.8 (2.13)	0.4837
Muscle bundle characteristics				
Bundle area (mm^2^)	0.37 (0.03)	0.36 (0.03)	0.42 (0.03)	0.3117
Fiber number per bundle	92.8 (12.2)	97.3 (12.2)	107 (9.96)	0.6051

^1^ Standard error of the mean.

**Table 3:** Comparison of sensory quality characteristics of the Hanwoo *Longissimus thoracis* muscle in groups categorized according to Warner-Bratzler shear force (WBS) values.

	WBS Value Group			*p*-Value
	Low	Medium	High	
Softness	6.02 ^a^ (0.23) ^1^	5.20 ^b^ (0.23)	4.67 ^c^ (0.25)	0.0012
Initial tenderness	5.92 ^a^ (0.24)	5.00 ^b^ (0.24)	4.55 ^b^ (0.26)	0.0016
Chewiness	5.59 ^a^ (0.25)	4.90 ^ab^ (0.25)	4.24 ^b^ (0.26)	0.0038
Rate of breakdown	5.50 ^a^ (0.22)	4.96 ^ab^ (0.22)	4.60 ^b^ (0.24)	0.0315
Amount of perceptible residue	5.68 ^a^ (0.21)	5.14 ^ab^ (0.21)	4.91 ^b^ (0.22)	0.0429
Overall tenderness	5.78 ^a^ (0.25)	4.94 ^b^ (0.25)	4.47 ^b^ (0.27)	0.0037
Juiciness	5.19 (0.21)	4.89 (0.21)	4.67 (0.22)	0.2365
Flavor intensity	5.62 (0.14)	5.66 (0.14)	5.49 (0.15)	0.7169
Off-flavor intensity	6.36 (0.18)	6.36 (0.18)	6.03 (0.19)	0.3641
Overall acceptability	5.71 ^a^ (0.25)	4.93 ^b^ (0.25)	4.49 ^b^ (0.27)	0.0065

^a–c^ Different superscript letters in the same row represent significant differences (*p* < 0.05). ^1^ Standard error of the mean.
